# Social inequalities in the use of contraceptives in adult women from Southern Brazil

**DOI:** 10.11606/S1518-8787.2019053000861

**Published:** 2019-03-27

**Authors:** Tonantzin Ribeiro Gonçalves, Heloísa Marquardt Leite, Fernanda Souza de Bairros, Maria Teresa Anselmo Olinto, Nêmora Tregnago Barcellos, Juvenal Soares Dias da Costa

**Affiliations:** I Universidade do Vale do Rio dos Sinos. Escola de Saúde. Programa de Pós-Graduação em Saúde Coletiva. São Leopoldo, RS, Brasil; II Universidade Federal do Rio Grande do Sul. Escola de Enfermagem. Departamento de Assistência e Orientação Profissional. Porto Alegre, RS, Brasil; III Universidade Federal de Ciências da Saúde de Porto Alegre. Departamento de Nutrição. Porto Alegre, RS, Brasil

**Keywords:** Contraception, methods, Socioeconomic Factors Health Status Disparities Contraceptive Prevalence Surveys, Sexual and Reproductive Health, Anticoncepção, métodos, Fatores Socioeconômicos Disparidades nos Níveis de Saúde Inquéritos sobre o Uso de Métodos Contraceptivos, Saúde Sexual e Reprodutiva

## Abstract

**OBJECTIVE:**

To describe the contraceptive methods used by adult women and the associated socioeconomic and demographic factors.

**METHODS:**

Population-based cross-sectional study with 20 to 49-year-old women from São Leopoldo, state of Rio Grande do Sul, in 2015. Three outcomes were considered to analyze the association with demographic and socioeconomic characteristics: use of oral contraceptive pills, tubal ligation and male condom. The crude prevalence ratios, stratified by age, and 95% confidence intervals (95%CI) were obtained using Poisson regression, taking the experimental error into account.

**RESULTS:**

A total of 736 women, aged from 20 to 49 years old, were evaluated. The prevalence of the use of oral contraceptive pills, tubal ligation and male condom were respectively 31.8% (95%CI 28.4–35.3), 11.1% (95%CI 9.0–13.6) and 10.9% (95%CI 8.7–13.3). In addition, 10.5% (n = 77) of the women reported making combined use of oral contraceptive pills and condom. In the stratified analysis, younger women with lower education level and from lower social classes reported less use of oral contraceptive pills. Tubal ligation was more prevalent among the lower social classes, but only in the age group from 30 to 39 years old. No differences were found in relation to male condom.

**CONCLUSIONS:**

The results indicated that differences persist in relation to contraception, which can be associated with both the difficulties of access to these inputs and the frailty of actions in reproductive health to achieve the needs and preferences of women who are more socially vulnerable.

## RESUMO

**OBJETIVO:**

Descrever os métodos contraceptivos utilizados e fatores demográficos e socioeconômicos associados em mulheres adultas.

**MÉTODOS:**

Estudo transversal de base populacional com mulheres de 20 a 49 anos de São Leopoldo, RS, em 2015. Foram considerados três desfechos para analisar a associação com características demográficas e socioeconômicas: uso de anticoncepcional oral, ligadura tubária e uso de preservativo masculino. Foram obtidas razões de prevalências, brutas e estratificadas por idade, e intervalos de confiança de 95% (IC95%) por meio de regressão de Poisson, levando em conta o erro de delineamento.

**RESULTADOS:**

Foram avaliadas 736 mulheres com idades entre 20 e 49 anos. A prevalência de uso de anticoncepcional oral, de ligadura tubária e de uso de preservativo masculino foram, respectivamente, 31,8% (IC95% 28,4–35,3), 11,1% (IC95% 9,0–13,6) e 10,9% (IC95% 8,7–13,3). Além disso, 10,5% (n = 77) das mulheres relataram fazer uso combinado de anticoncepcional oral e preservativo masculino. Na análise estratificada, as mulheres mais jovens, de menor escolaridade e classe econômica mais baixa relataram menor uso de anticoncepcional oral. Já a ligadura tubária foi mais prevalente entre as de classe econômica mais baixa, mas apenas na faixa etária de 30 a 39 anos. Não foram encontradas diferenças quanto ao preservativo masculino.

**CONCLUSÕES:**

Os resultados indicaram que ainda persistem diferenças quanto à contracepção, o que pode se relacionar tanto a dificuldades no acesso a esses insumos como a fragilidades das ações em saúde reprodutiva para atingir as necessidades e preferências das mulheres em maior vulnerabilidade social.

## INTRODUCTION

In the context of sexual and reproductive rights, individuals should be able to have a sex life that is both satisfying and safe, as well as the freedom to decide if, when and how often they wish to have children^1^. The access to information and contraceptive methods should be ensured by the State and by the healthcare system, to promote the responsible and equitable exercise of these rights. This conception was consolidated by the Programme of Action of the International Conference on Population and Development held in Cairo in 1994^1^. In Brazil, the Program of Full Assistance to Women's Health (PAISM), launched in 1983 by the Ministry of Health, established an important and pioneering milestone by incorporating the promotion of citizenship and individual empowerment in reproductive planning actions^2^.

The world trend is reducing unmet family planning needs. The use of contraceptive methods increased from 54.8% in 1990 to 63.3% in 2010^3^. Despite the different regional patterns, sterilization was still the most widely used method in developing countries in 2012, followed by the intrauterine device (IUD), oral contraceptive pills (OCP) and male condom^4^. Asia and Latin America experienced the greatest declines in sterilization between 2003 and 2012, corresponding to 9% and 15%, respectively, with consequent increase in the use of medium and long-term barrier and hormonal methods (injection and implants)^4^. In Brazil, between 1990 and 2010, the percentage of unmet family planning needs decreased from 11.6% to 7.4%, becoming the fourth lowest rate in South America^3^.

The main methods used by Brazilian women in reproductive age have been OCP and tubal ligation (TL), followed by male condom. Recent data from the National Survey on Access, Use and Promotion of the Rational Use of Medicines indicate the prevalence of use of OCP (28.2%) and contraceptive injection (4.5%) in 15 to 49-year-old non-pregnant women residing in urban areas^5^. The Southern region of the country showed higher prevalence of use of OCP (37.5%), and most of the women paid for the medicine, as opposed to users of contraceptive injection, which was most accessed through the public services^5^. Corroborating these findings, population studies carried out in the municipalities of Rio Grande do Sul showed high prevalence of use of OCP, between 48.8%^6^ and 55.4%^7^. The prevalence of TL reported in Brazilian studies ranged from 18.7%^6^ to 22%^7^ ^,^ ^8^, while the rate of use of condom as contraceptive method remained lower, between 10% and 17%^6^ ^–^ ^8^.

Despite the advances, the full implementation of PAISM encountered numerous obstacles, and social inequalities in the access and use of modern contraceptives have not ceased to exist^9^ ^,^ ^10^. Data from the 2006 National Survey of Children and Women's Demography and Health (PNDS) showed that the percentage of 15 to 44-year-old women who did not use any method was still nearly twice that of higher-class women for those in the lower classes^8^. Thus, although fertility rates have decreased dramatically in recent decades, this reduction reached different population strata unequally, with little impact among the youth under 20 years old and among black and mixed-race women with lower education level^11^.

Therefore, it is important to monitor social inequality indicators when assessing the use of contraceptive methods from specific regional realities. These data may guide the planning of actions and policies to ensure sexual and reproductive rights from the perspective of equity and diversity, reducing barriers of access to different contraceptive methods according to the women's preferences and needs. Thus, the objective of this study was to describe the contraceptive methods used by women with an active sexual life, aged from 20 to 49 years old. In addition, the associated demographic and socioeconomic characteristics were analyzed, and the results were compared with data from a population study conducted with women from the same city eleven years before.

## METHODS

This research is part of a population-based cross-sectional study with a representative sample of women aged from 20 to 69 years old, living in the urban area of the city of São Leopoldo, RS, whose data collection occurred between February and October 2015. The present study included all 20 to 49-year-old women and the data were compared to those of another investigation with the same methodological features, conducted in 2003^6^.

The municipality of São Leopoldo is located in the region of Vale do Rio dos Sinos, Metropolitan Region of Porto Alegre, with a total population of 214,087 people. In the 2010 census, 71,564 women aged between 20 and 69 years old were recorded in the city's urban area, representing 32% of the total population^12^.

Sample size was calculated from the prevalence of various outcomes related to women's health and contraception. We opted for the result that required greater sample size, i.e., the frequency of delayed cervical screening^13^. A 2.0 risk ratio between women with lower and higher education level (non-exposed – 15 years or more of education) was found considering 80% power and 95% confidence level. Sample size was increased by 10% to contemplate possible losses and refusals and by 15% for the control of confounding factors. Thus, it was estimated that a sample made up of 1,281 women in a total of 1,613 households would be necessary.

The sample was systematic. The 371 census tracts in the urban region of São Leopoldo were sorted in descending order, starting from the tract with greater “value of nominal monthly income of people aged 10 years old or older (with or without an income),” according to the Brazilian Institute of Geography and Statistics (IBGE)^12^. Then, 45 census tracts were randomly chosen. In a second stage, one block in each sector and, later, a street corner for the beginning of the interviews were randomly chosen. From the initial corner, the first house on the left of who was facing it was selected, two houses were skipped and the next house was selected (thus, the fourth house), totaling 36 households per tract. In the case of absence of the residents, the interviewer made two more attempts at different days and times. If the women to be interviewed were not at home, a new visit was scheduled.

Every participant filled a standardized and pre-tested questionnaire including demographic, behavioral, psychosocial and socioeconomic variables, as well as data on the use of health services. Pregnant women or those in no condition to answer the survey were not interviewed. The interviewers were undergraduate and graduate students trained for the instrument's implementation. A pilot study was conducted in a sector that was not included in the survey to complete the training of the interviewers, as well as to test the instrument and the survey's logistics.

The data collection's quality control was conducted by phone or home visit with a random sample of 10% of the participants. The control instrument included variables that would not change in the short term.

The prevalence of use of contraceptive methods was investigated based on the same questions of the study conducted in 2003^6^. The women were asked if they had had intercourse in the last year and which contraceptive method(s) they used. The women could report the simultaneous use of more than one contraceptive method or dual protection (use of male condom and another method). Three contraceptive methods were included in the analyses: OCP, TL and male condom. For the first method, women who used OCP in isolation and combined with a male condom were considered. For the second method, those who reported having been submitted to the TL procedure were considered. For the third method, women who declared using a condom in isolation or combined with other methods, such as IUDS, ring and OCP, were considered.

The exposure variables were age, self-reported skin color, social class according to the Brazilian Association of Research Companies (ABEP)^a^ and the women's education level. The age variable was analyzed in age ranges: 20 to 29 years old; 30 to 39 years old; 40 to 49 years old. As for skin color, women who declared themselves as white, mixed race and black were considered. Only seven women self-reported themselves as indigenous and eight as yellow; the 15 were excluded. The social class according to ABEP's classification considered the possession of certain material goods, education level of the head of the family and the number of people employed by the family. For the analysis, the class was organized in three categories: A+B; C and D+E. Education level was obtained in number of complete years of education and categorized into five levels: zero to four years; five to seven years; eight to 10 years; 11 to 14 years; 15 years or more.

The double-entry of the data was conducted with the aid of Epidata 3.1 (Centers for Disease Control and Prevention, Atlanta, United States). The analysis was performed in Stata 13.0 (Stata Corp., College Station, United States) to describe the prevalence of the non-use and use of the contraceptive methods. Initially, the prevalence ratios were calculated (PR) with their respective 95% confidence intervals (95%CI) and the associations of the use of OCP, TL and condom with the demographic and socioeconomic variables were analyzed. Finally, the analyses were stratified by age, separately investigating the associations with the skin color variable and socioeconomic characteristics for each of the groups: OCP, TL and male condom. The raw and stratified results were obtained using Poisson regression, taking the design error into account through the svy command in Stata.

The research project was approved by the Research Ethics Committee of the University of Vale do Rio dos Sinos (Protocol 653,394, of 20 May, 2014), and all the participants signed an informed consent form.

## RESULTS

Of the 1,281 women visited, 1,128 were interviewed and 153 (11.94%) were classified as losses and refusals. Of the 1,128 women included in the study, 736 were up to 49 years old, of which 54 (7.3%) declared not having had intercourse in the year preceding the interview, 83 (11.3%) did not use contraception, 22 (3%) were sterile and 577 (78.4%) used some kind of contraceptive method.

As for the contraceptive methods adopted, 234 women reported using OCP alone (31.8%; 95%CI 28.4–35.3), 82 reported TL (11.1%; 95%CI 9.0–13.6) and 80 (10.9%; 95%CI 8.7–13.3) used male condom exclusively. It is worth noting that 10.5% (n = 77) reported the combined use of OCP and male condom. In this way, of the women who reported using male condom (n = 163), 77 (47.2%) also reported the use of OCP.

When comparing the use of contraception in 2003 and 2015, there was a small decrease in the number of women who did not use contraceptives in the extreme socioeconomic classes, A+B and D+E. A slight increase in the percentage of use of OCP (in isolation and combined with condom) was noted in class A+B, reaching 49.5% in 2015. As for TL, its percentage showed a mild decrease, which was more pronounced in class A+B and D+E women. In 2015, the isolated use of condom decreased in almost all classes; however, when used in combination with OCP, it became the second most widely used method among the female population of São Leopoldo. An increase in the use of injectable contraception and vasectomy, especially in classes C and D+E (Table 1), was also noted.

**Table 1:** Contraceptive methods used by women from 20 to 49 years old of São Leopoldo, state of Rio Grande do Sul, according to social class, in 2003 (n = 672) and in 2015 (n = 736).

Contraceptive methods		Social class			Total
		A+B	C	D+E	
2003					
	Does not use	33(14.9)	33 (12.0)	32 (18.1)	98 (14.6)
	Oral contraceptive pills	77 (34.7)	126 (45.7)	78 (44.1)	281 (41.8)
	Oral contraceptive pills and condom	0 (0.0)	1 (0.4)	0 (0.0)	1 (0.1)
	Tubal ligation	38 (20.1)	38 (13.8)	32 (22.1)	108 (16.1)
	Male condom	35 (15.8)	42 (15.2)	23 (13.0)	100 (14.9)
	Injection	5 (2.3)	10 (3.6)	4 (2.3)	19 (2.8)
	Vasectomy	7 (3.2)	4 (1.4)	1 (0.6)	12 (1.8)
	IUD	18 (8.1)	17 (6.2)	7 (4.0)	42 (6.3)
	Other natural methods^a^	5 (2.3)	2 (0.7)	0 (0.0)	7 (1.0)
	Male condom and others^b^	0	1 (0.4)	0 (0.0)	1 (0.1)
	Other barrier methods^c^	2 (0.9)	1 (0.4)	0	3 (0.4)
2015					
	Does not use	26 (9.7)	45 (11.8)	12 (14.5)	83 (11.3)
	Sterile	5 (1.9)	14 (3.7)	3 (3.6)	22 (3.0)
	No sexual relations in the last year	14 (5.2)	32 (8.4)	8 (9.6)	54 (7.3)
	Oral contraceptive pills	96 (36.0)	121 (31.6)	16 (19.3)	234 (31.8)
	Oral contraceptive pills and condom	36 (13.5)	33 (8.6)	6 (7.2)	77 (10.5)
	Tubal ligation	26 (9.7)	45 (11.8)	11 (13.3)	82 (11.1)
	Male condom	30 (11.2)	39 (10.2)	11 (13.3)	80 (10.9)
	Injection	5 (1.9)	18 (4.7)	11 (13.3)	34 (4.6)
	Vasectomy	12 (4.5)	16 (4.2)	2 (2.4)	30 (4.1)
	IUD	12 (4.5)	10 (2.6)	1 (1.2)	23 (3.1)
	Other natural methods^a^	2 (0.8)	6 (1.6)	0 (0.0)	8 (1.1)
	Male condom and others^b^	1 (0.4)	4 (1.0)	1 (1.2)	6 (0.8)
	Other barrier methods^c^	2 (0.8)	0 (0.0)	1 (1.2)	3 (0.4)

a Coitus interruptus, monitoring of cervical mucus and rhythm method.

b Other barrier methods and/or morning-after pill.

c Intravaginal Ring and diaphragm.

In relation to the prevalence in the use of OCP, it decreased with age as well as with social class and education level. Prevalence was 46% and 63% lower in class D+E women and in those with zero to four years of education, compared to those in classes A+B and with 15 years or more of education, respectively. The prevalence of use of OCP did not differ significantly between the skin color categories (Table 2).

**Table 2:** Prevalence, prevalence ratios (PR) and 95% confidence intervals (95%CI) of use of oral contraceptive pills, tubal ligation and male condom according to socioeconomic and demographic variables in adult females of São Leopoldo, state of Rio Grande do Sul, 2015. (n = 736)

Variable		n (%)	Prevalence (%)	PR	95%CI	p
Oral contraceptive pills						
Age group (years)						< 0.001*
	20–29	216 (29.4)	129 (59.7)	1		
	30–39	244 (33.2)	115 (47.1)	0.79	0.65–0.96	
	40–49	276 (37.5)	67 (24.3)	0.41	0.31–0.53	
Skin color						0.965
	White	536 (74.8)	228 (42.5)	1		
	Mixed	120 (16.7)	50 (41.7)	0.98	0.75–1.28	
	Black	61 (8.5)	25 (41.0)	0.96	0.70–1.33	
Social class						0.003*
	A or B	267 (36.4)	132 (49.4)	1		
	C	383 (52.3)	154 (40.2)	0.81	0.70–0.94	
	D or E	83 (11.3)	22 (26.5)	0.54	0.39–0.75	
Education level (years)						< 0.001*
	15 or more	74 (10.1)	44 (59.5)	1		
	11 to 14	287 (39.0)	143 (49.8)	0.84	0.65–1.09	
	8 to 10	142 (19.3)	60 (42.3)	0.71	0.54–0.94	
	5 to 7	151 (20.5)	46 (30.5)	0.51	0.37–0.70	
	0 to 4	82 (11.1)	18 (22.0)	0.37	0.24–0.57	
Tubal ligation						
Age group (years)						< 0.001*
	20–29	216 (29.4)	5 (2.3)	1		
	30–39	244 (33.2)	22 (9.0)	3.90	1.45–10.5	
	40–49	276 (37.5)	55 (19.9)	8.61	3.76–19.7	
Skin color						0.541
	White	536 (74.8)	58 (10.8)	1		
	Mixed	120 (16.7)	16 (13.3)	1.23	0.77–1.97	
	Black	61 (8.5)	5 (8.2)	0.76	0.30–1.93	
Social class						0.532
	A or B	267 (36.4)	26 (9.7)	1		
	C	383 (52.3)	45 (11.8)	1.21	0.76–1.92	
	D or E	83 (11.3)	11 (13.3)	1.36	0.73–2.52	
Education level (years)						< 0.001*
	15 or more	74 (10.1)	2 (2.7)	1		
	11 to 14	287 (39.0)	20 (7.0)	2.57	0.57–11.7	
	8 to 10	142 (19.3)	21 (14.8)	5.47	1.23–24.3	
	5 to 7	151 (20.5)	21 (13.9)	5.15	1.14–23.2	
	0 to 4	82 (11.1)	18 (22.0)	8.12	1.83–36.1	
Male condom						
Age group (years)						< 0.001*
	20–29	216 (29.4)	66 (30.6)	1		
	30–39	244 (33.2)	59 (24.2)	0.79	0.61–1.02	
	40–49	276 (37.5)	38 (13.8)	0.45	0.31–0.66	
Skin color						0.174
	White	536 (74.8)	119 (22.2)	1		
	Mixed	120 (16.7)	30 (25.0)	1.13	0.84–1.51	
	Black	61 (8.5)	8 (13.1)	0.59	0.30–1.18	
Social class						0.442
	A or B	267 (36.4)	67 (25.1)	1		
	C	383 (52.3)	76 (19.8)	0.79	0.55–1.14	
	D or E	83 (11.3)	18 (21.7)	0.86	0.54–1.37	
Education level (years)						< 0.001*
	15 or more	74 (10.1)	22 (29.7)	1		
	11 to 14	287 (39.0)	79 (27.5)	0.93	0.62–1.38	
	8 to 10	142 (19.3)	29 (20.4)	0.69	0.45–1.06	
	5 to 7	151 (20.5)	21 (13.9)	0.47	0.28–0.79	
	0 to 4	82 (11.1)	12 (14.6)	0.49	0.26–0.93	

* Linear trend test.

As for TL, prevalence increased with age. Linear inverse association was found with education level, i.e., prevalence was higher the lower the education level. In women with zero to four years of education, the prevalence of TL was eight times higher when compared to those who reported 15 years or more of education. However, the prevalence of TL did not differ significantly between the skin color and social class categories (Table 2).

Regarding the use of male condom in isolation and combined with some other method, it was noted that women aged from 40 to 49 years old reported lower use than those aged from 20 to 29 years old. As for education level, women with up to seven years of education also used male condoms less. No significant association was verified for the use of male condom with skin color and social class (Table 2).

In the stratified analysis, for the use of OCP, there were significant differences in the age groups from 20 to 29 years old and from 30 to 39 years old (Table 3). In these age groups, the D+E class women with lower education level used OCP less.

**Table 3:** Prevalence, prevalence ratios (PR) and 95% confidence intervals (95%CI) for use of oral contraceptive pills, tubal ligation and male condom according to socioeconomic and demographic variables in adult females of São Leopoldo, state of Rio Grande do Sul, 2015. (n = 736)

Variable		n (%)	Prevalence %	PR	95%CI	p
20–29 years						
Skin color						0.701
	White	155 (74.2)	61.3	1		
	Mixed	39 (18.7%)	53.9	0.88	0.63–1.22	
	Black	15 (7.2)	53.3	0.87	0.52–1.45	
Social class						0.010*
	A or B	71 (33.0)	69.0	1		
	C	116 (53.9)	58.6	0.85	0.68–1.06	
	D or E	28 (13.0)	39.3	0.57	0.35–0.92	
Education level (years)						0.004*
	15 or more	16 (7.4)	87.5	1		
	11 to 14	112 (51.9)	62.5	0.71	0.55–0.92	
	8 to 10	53 (24.5)	58.5	0.67	0.51–0.87	
	5 to 7	30 (13.9)	43.3	0.50	0.30–0.81	
	0 to 4	5 (2.3)	20.0	0.23	0.04–1.43	
30–39 years						
Skin color						0.504
	White	185 (77.1)	46.5	1		
	Mixed	28 (11.7)	57.1	1.23	0.82–1.85	
	Black	27 (11.2)	40.7	0.88	0.52–1.48	
Social class						0.002*
	A or B	89 (36.5)	58.4	1		
	C	127 (52.1)	44.9	0.77	0.57–1.03	
	D or E	28 (11.5)	21.4	0.37	0.16–0.84	
Education level (years)						0.008*
	15 or more	40 (16.4)	60.0	1		
	11 to 14	93 (38.1)	52.7	0.88	0.59–1.31	
	8 to 10	48 (19.7)	41.7	0.69	0.43–0.93	
	5 to 7	40 (16.4)	37.5	0.63	0.42–0.99	
	0 to 4	23 (9.4)	30.4	0.51	0.32–1.07	
40–49 years						
Skin color						0.662
	White	196 (73.1)	23.9	1		
	Mixed	53 (19.8)	24.5	1.02	0.57–1.85	
	Black	19 (7.1)	31.6	1.32	0.72–2.43	
Social class						0.301
	A or B	107 (39.1)	28.9	1		
	C	140 (51.1)	20.7	0.71	0.46–1.10	
	D or E	27 (9.9)	18.5	0.64	0.21–1.97	
Education level (years)						0.441
	15 or more	18 (6.5)	33.3	1		
	11 to 14	82 (29.7)	29.3	0.88	0.37–2.07	
	8 to 10	41 (14.9)	21.9	0.66	0.29–1.50	
	5 to 7	81 (29.4)	22.2	0.67	0.33–1.34	
	0 to 4	54 (19.6)	18.5	0.56	0.21–1.49	

* Linear trend test.

Differently from the crude analysis, the stratified analysis indicated that women aged from 30 to 39 years old in social class D+E were almost five times more likely to have been submitted to tubal ligation than those in class A+B. The stratification of TL by age was not possible for education level and skin color, as in the age groups from 20 to 29 years old and from 30 to 39 years old there were no women with 15 years or more of education and black skin that had been submitted to the procedure (Table 4).

**Table 4:** Prevalence, prevalence ratios (RP) and 95% confidence intervals (95%) for tubal ligation according to social class stratified by age in adult females of São Leopoldo, state of Rio Grande do Sul, 2015. (n = 736)

Variable		n (%)	Prevalence %	PR	95%CI	p
20–29 years						
Social class						0.788
	A or B	71 (33.0)	1.4	1		
	C	116 (53.9)	2.6	1.84	0.19–17.3	
	D or E	28 (13.0)	3.6	2.54	0.15–41.7	
30–39 years						
Social class						0.017*
	A or B	89 (36.5)	4.5	1		
	C	127 (52.1)	9.5	2.10	0.65–6.79	
	D or E	28 (11.5)	21.4	4.77	1.31–17.3	
40–49 years						
Social class						0.828
	A or B	107 (39.1)	19.6	1		
	C	140 (51.1)	21.4	1.09	0.63–1.89	
	D or E	27 (9.9)	14.8	0.75	0.25–2.32	

* Linear trend test.

For the use of male condoms, there was no significant association with social class in the analysis stratified by age. This analysis was not possible for education level and skin color, because in the age groups from 20 to 29 years old and from 30 to 39 years old, no woman with zero to four years of education reported use of male condom, as in the range from 40 to 49 years old for black skin color (Table 5).

**Table 5:** Prevalence, prevalence ratios (RP) and 95% confidence intervals (95%) for use of male condoms according to social class stratified by age in adult females of São Leopoldo, state of Rio Grande do Sul, 2015. (n = 736)

Variable		n (%)	Prevalence %	PR	95%CI	p
20–29 years						
Social class						0.132
	A or B	71 (33.0)	39.4	1		
	C	116 (53.9)	25.9	0.66	0.42–1.02	
	D or E	28 (13.0)	25.0	0.63	0.29–1.37	
30–39 years						
Social class						0.217
	A or B	89 (36.5)	30.3	1		
	C	127 (52.1)	21.3	0.70	0.45–1.09	
	D or E	28 (11.5)	17.9	0.59	0.25–1.41	
40–49 years						
Social class						0.192
	A or B	107 (39.1)	11.2	1		
	C	140 (51.1)	13.6	1.21	0.58–2.54	
	D or E	27 (9.9)	22.2	1.98	0.93–4.24	

## DISCUSSION

The present study sought to describe the contraceptive methods used by women aged from 20 to 49 years old with an active sex life from São Leopoldo, state of Rio Grande do Sul, in 2015, identifying the associated demographic and socioeconomic aspects and comparing the temporal trends in the same population stratum. In line with the national and international literature, it was possible to note a decrease in the number of women who did not use any method, an increase in the use of OCP (combined or in isolation) and decrease in the use of TL between 2003 and 2015^3^ ^,^ ^4^ ^,^ ^8^ ^,^ ^14^. Overall, the prevalence of OCP in the women of São Leopoldo in 2015 remained well above the recent averages found for Brazil^5^, for the southern region of the country^5^ and for South American countries^4^.

Despite the massive use of OCP in this population, comparing the data of 2003 and 2015, there was a discreet tendency towards diversification of contraceptive methods over time, with increase in the use of vasectomy and injectable contraceptive, as well as in the so-called dual protection with the use of condom. These data may reflect the increase in the access to and offer of different methods, in addition to a greater concern with the prevention of sexually transmitted infections. At the same time, the percentage of women subjected to TL decreased between 2003 and 2015, especially among the highest and lowest social classes, which may relate to both this greater diversity and possibility of choice of contraceptive methods and the restrictions and unmet demands associated with the implementation of the family planning law^15^.

On the other hand, the use of the IUD had low prevalence in the sample. Its use decreased between 2003 and 2015 in all social classes, representing less than half the percentage reported for South American countries^4^. There were also no reports of the use of long-term reversible contraceptives, such as subdermal implants, which are not yet offered by SUS. In the city of São Leopoldo, the placement of the IUD is centralized in only one of SUS's reference centers, which is also responsible for TL. It is speculated that the greater ease of access to contraceptive injection (prescribed and dispensed in the basic units)^16^ and vasectomy (for which there is no waiting list) make the IUD a less viable option for women. The need for placement of the IUD is similar to that of TL. As it is a reversible method, with high efficiency, low cost and few side effects, access to the IUD in the healthcare network must be facilitated so that it is an actual alternative for women considering the massive use of OCP, which may be contraindicated in some situations^17^.

The findings of the study revealed that important social inequalities persisted regarding the use of contraceptive methods, which may be related both to difficulties in access and to the frailty of actions in reproductive health to achieve the needs and preferences of women who are more socially vulnerable^18^ ^,^ ^19^. The use of OCP was significantly lower among younger women from the lower social classes with lower education level, characterizing this group as more vulnerable to unwanted pregnancies, as found in low and medium-income countries^20^. This can be associated with the lack of information on family planning and contraceptives due to the greater difficulty of access to health services, seeing as the sexual life of young, unmarried people is socially condemned^18^ ^,^ ^21^.

Unlike the findings about the use of OCP, TL was more prevalent among women aged from 30 to 39 years old from the lower social classes, corroborating what was found in the same stratum of the population of São Leopoldo in 2003^6^. These results go against the national data of PNDS in 2006^8^, which pointed to a more uniform distribution of TL between the social classes even after adjustment for age, number of children and place of residence. As for education level, no differences were found, unlike in international studies^22^. In this sense, regional dynamics may have a different impact on how social inequalities present themselves. However, it must be noted that it was not possible to assess the quality of contraception (i.e., use of contraception before having children and number of unwanted pregnancies), which would have been a more sensitive indicator of reproductive control among women of the lower social classes^8^.

The isolated or combined use of condoms was reported by less than a quarter of all women in the study, similarly to what was found among sexually active women of São Leopoldo in 2003^23^ and among Brazilian women in 2008^24^. In addition, it was not possible to detect any differences related to the women's social class, like was found by the national American 2011-2013 survey on family growth^22^. The low frequency of use of condoms by women denotes the persistence of this population's vulnerability to infection by HIV^25^. Therefore, it would be important to strengthen strategies to promote the use of condoms, favoring the different social contexts and sexual practices^26^.

The present study also showed no differences in the use of contraceptive methods with respect to the women's skin color. American population studies, on the other hand, have found differences for unmet contraceptive needs, use of OCP and reversible long-term methods^27^ ^,^ ^28^, the data about TL being controversial^22^ ^,^ ^27^ ^,^ ^28^. In addition, it is important to note that the processes that operate the institutional racism in the distribution of services, benefits and opportunities are subtle^29^. This daily production of iniquities under the racial point of view has been seen, for example, in indicators of prenatal and childbirth care in Brazil^30^, and it may be more difficult to identify them in the use of contraceptive methods, which are often acquired by the women themselves^5^ as an alternative to the difficulty of access through SUS.

The transversal nature of the study is a limitation to understanding the processes through which social inequalities affect the use of contraceptive methods, and it is important to monitor the women's reproductive trajectories. In this study, it was not possible to evaluate the conditions of access, the situations of contraindication to contraceptive methods or even the quality of contraception, aspects that could also indicate inequalities in health. However, considering the lack of recent studies on contraception with representative samples, these findings are important to monitor trends and iniquities, assisting the planning of actions focused on women's health in this community.

## Footnotes

a Associação Brasileira de Empresas de Pesquisa. Critério de Classificação Econômica Brasil. São Paulo: ABEP; 2016 [cited 2018 Dec 12]. Available from: http://www.abep.org/criterio-brasil
